# Improving Prescribing Documentation in a Community Learning Disability Team: A Clinical Audit and SOP (Standard Operating Procedure) Development

**DOI:** 10.1192/bjo.2026.11668

**Published:** 2026-06-30

**Authors:** Shriya Mathur

**Affiliations:** Leeds and York Partnership NHS Foundation Trust, Leeds, United Kingdom

## Abstract

**Aims:**

Background

In April 2025, city-wide changes to General Practitioner (GP) prescribing increased prescribing activity within the Community Learning Disability Team (CLDT). This audit evaluated the quality and consistency of prescribing documentation to determine whether current practice met local standards and to identify areas requiring improvement.

Aims

1. Assess adherence to documentation standards for prescriptions recorded in the electronic health record (Care Director), clinic register, and clinical notes.

2. Describe prescribing patterns across the team.

3. Develop a Standard Operating Procedure (SOP) to address gaps identified through the audit.

**Methods:**

Records of 297 service users receiving prescriptions between 01/04/2024 and 31/05/2025 were reviewed. Outcomes included whether prescriptions were uploaded to Care Director (scan or photograph), whether entries were completed in the clinic register, whether prescribing was documented elsewhere (case notes or letters), and the site of prescription issue. Prescribing patterns were summarised.

**Results:**

A total of 319 prescriptions were issued to 147 patients. Of these, 65% (208/319) were uploaded to Care Director–half scanned and half photographed. Only 19% (58/300; clozapine excluded) were entered into the clinic register. Prescribing was documented elsewhere in 98% (314/319). Documentation occurred frequently but was inconsistent in format, location, and completeness.

**Conclusion:**

Conclusions

The audit identified frequent but inconsistent documentation. The SOP provides a structured framework to improve accuracy, consistency, and audit-ability, with re-audit recommended at 6–12 months.

Actions

Based on the identified gaps, a SOP was developed. Key components included a scanfirst requirement, defined legibility standards, mandatory casenote entry confirming medication and dose, creation of a centralised electronic tracking register, quarterly monitoring, and data protection guidance.

